# The first recombinant human coagulation factor VIII of human origin: human cell line and manufacturing characteristics

**DOI:** 10.1111/j.1600-0609.2012.01804.x

**Published:** 2012-06-15

**Authors:** Elisabeth Casademunt, Kristina Martinelle, Mats Jernberg, Stefan Winge, Maya Tiemeyer, Lothar Biesert, Sigurd Knaub, Olaf Walter, Carola Schröder

**Affiliations:** 1 OctapharmaMunich, Germany*; 2 OctapharmaStockholm, Sweden; 3 OctapharmaFrankfurt, Germany; 4 OctapharmaLachen, Switzerland

**Keywords:** Haemophilia A, recombinant FVIII, immunogenicity, HEK 293 F human cell line, pathogen safety

## Abstract

**Introduction:**

Since the early 1990s, recombinant human clotting factor VIII (rhFVIII) produced in hamster cells has been available for haemophilia A treatment. However, the post-translational modifications of these proteins are not identical to those of native human FVIII, which may lead to immunogenic reactions and the development of inhibitors against rhFVIII. For the first time, rhFVIII produced in a human host cell line is available.

**Aim:**

We describe here the establishment of the first human production cell line for rhFVIII and the manufacturing process of this novel product.

**Methods and results:**

A human cell line expressing rhFVIII was derived from human embryonic kidney (HEK) 293 F cells transfected with an FVIII expression plasmid. No virus or virus-like particles could be detected following extensive testing. The stringently controlled production process is completely free from added materials of animal or human origin. Multistep purification employing a combination of filtration and chromatography steps ensures the efficient removal of impurities. Solvent/detergent treatment and a 20 nm pore size nanofiltration step, used for the first time in rhFVIII manufacturing, efficiently eliminate any hypothetically present viruses. In contrast to hamster cell-derived products, this rhFVIII product does not contain hamster-like epitopes, which might be expected to be immunogenic.

**Conclusions:**

HEK 293 F cells, whose parental cell line HEK 293 has been used by researchers for decades, are a suitable production cell line for rhFVIII and will help avoid immunogenic epitopes. A modern manufacturing process has been developed to ensure the highest level of purity and pathogen safety.

Haemophilia A is an X-linked hereditary disease caused by factor VIII (FVIII) deficiency 1 which, if left untreated, leads to haemorrhages, mostly into muscles and joints 2, and consequently to arthropathy and severe morbidity. Factor VIII replacement, either on-demand or as prophylaxis, is the mainstay of current therapy for severe disease (defined as FVIII:C <1%); prophylactic treatment has been shown to reduce the number of bleeding episodes and the risk of permanent joint damage 3.

Recombinant human (rh)FVIII products have been available for 20 years; so far, all proteins have been produced in either Chinese hamster ovary (CHO) or baby hamster kidney cells (BHK) 4–6. While these products have been able to alleviate concerns about supply shortages and show good pathogen safety profiles, they display a non-human pattern of post-translational modifications (PTMs).

FVIII is subjected to multiple PTMs, especially glycosylations, and is considered the largest and most complex marketed protein produced by recombinant DNA technology to date 7. Incorrect reproduction of these PTMs in a non-human expression system may trigger immune reactions and lead to the formation of inhibitors against FVIII, which may render FVIII replacement therapy ineffective. Inhibitor development is a severe complication that is frequently observed in haemophilia A therapy. Two systematic reviews evaluating data from previously untreated patients with severe haemophilia A receiving currently marketed recombinant human clotting factor VIII (rhFVIII) products from hamster cell lines reported a cumulative risk of inhibitor development of up to 39% 8 and a rate of inhibitor development of 34.5% 9, respectively. Inhibitor development occurs throughout life in haemophilia patients 10 and causes considerable distress to the patient and equally considerable costs to healthcare systems 11–12. It is one of the main concerns regarding FVIII therapy, both from a patient's and a physician's perspective 13–14.

Recently, a cell line of human origin (human-cl) has been used for the first time to produce rhFVIII. Human-cl rhFVIII is characterised in detail elsewhere (Sandberg H, Kannicht C, Stenlund P, Dadaian M, Oswaldsson U, Cordula C, Walter O, and Kannicht C, Ramström M, Kohla G, Tiemeyer M, Casademunt E, Walter O, Sandberg H, manuscripts submitted for publication) and represents an innovative improvement in the category of rhFVIII preparations.

The human embryonic kidney cell line HEK 293 has been widely employed as an expression host in scientific research (a search with the key term ‘protein expression in HEK 293 cells’ identifies 5276 references), but has rarely been used for the commercial production of recombinant proteins. The technical advantages of this cell line include its robust growth pattern, easy maintenance, and high efficiency of transfection and protein production 15–16. Successful attempts to produce active rhFVIII in HEK 293-based cell lines in laboratory scale have recently been reported 17.

Until a few years ago, a HEK 293-derived cell line pre-adapted to serum-free culture conditions was not available. Although HEK 293 cells could in principle have been adapted to grow in serum-free medium 15, the transfer of clones to serum-free medium is an extremely long and inefficient process whereby many clones do not survive. Most clones also produce lower levels of recombinant protein after the transfer to serum-free medium. These issues prevented the use of HEK 293 cells for commercial-scale protein production. However, in 2003, HEK 293 cells were finally adapted to grow in suspension culture and in the absence of serum by Invitrogen, and this cell line was called HEK 293 F 18. Octapharma chose HEK 293 F as a host cell line and subsequently developed all the necessary methods for industrial recombinant protein production in these cells, based on extensive previous experience with the parental HEK 293 cells as well as with CHO and BHK hamster cell lines. The successful adaptations of methodology to the unique characteristics of HEK 293 F cells enabled the use of this cell line as a production cell line. The human-cl rhFVIII production cell line was generated by Octapharma by transfection of HEK 293 F cells with a human FVIII cDNA.

The main reason for using a human cell line is the presence of the translational machinery and all the subcellular compartments necessary to generate correctly folded and fully active FVIII 19, with an authentic human pattern of PTMs. This is of particular importance as PTMs have been demonstrated to impact on rhFVIII activity (Sandberg H, Kannicht C, Stenlund P, Dadaian M, Oswaldsson U, Cordula C, Walter O, manuscript submitted for publication). PTMs are also a crucial determinant of the immunogenic potential of rhFVIII. In this context, it is important to note that with a human host system, any potential residual traces of host cell protein will be human in nature and therefore less immunogenic than rodent host cell proteins. Additionally, the production process of human-cl rhFVIII has been optimised to exclude any raw materials of animal origin (including rodent antibodies, which are frequently used for monoclonal antibody purification), minimising the risk of allergic reactions to animal proteins. The aim of this article is to outline the development, production and purification of this first rhFVIII produced in human cells.

## Materials and methods

### Development of the production cell line

HEK 293 F cells (Invitrogen, available via LuBioScience, Lucerne, Switzerland) were cultured in FreeStyle™ 293 Expression medium (Invitrogen, available via LuBioScience) and stably transfected with an Octapharma proprietary expression plasmid carrying recombinant B-domain-deleted human FVIII cDNA. The vector used to generate the expression plasmid carries a promoter that is highly active in eukaryotic cells, as well as a transcription termination signal that enhances the stability of the mRNA. In preliminary experiments to compare several backbones, this vector showed the highest level of rhFVIII expression in HEK 293 F cells.

Transfectants were selected in hygromycin-containing medium and analysed for levels and quality of secreted FVIII. Subcloning yielded a clone with superior growth properties, stability, productivity levels and quality of the secreted FVIII, which was expanded and cryopreserved as a research cell bank (RCB). This bank was used to generate a master cell bank (MCB), from which the working cell bank (WCB) was produced. MCB and WCB were stored in the vapour phase of liquid nitrogen at two different geographical sites. Production cells were obtained by expansion of the WCB.

All manipulations were performed in the absence of any animal-derived components; production cells were cultured in a proprietary low-protein medium without any additives of human or animal origin (original source data, Octapharma).

### Isoenzyme analysis

Isoenzyme analysis is based on the existence of enzymes that possess similar or identical specificity, but which differ in their molecular structure (isoenzymes). Isoenzymes present in cell lysates will produce a species-specific migration pattern following agarose gel electrophoresis 20.

Isoenzyme analysis was performed using the AuthentiKit™ system (Innovative Chemistry Inc., Marshfield, MA, USA) according to the manufacturer's instructions. Enzymes analysed were nucleoside phosphorylase (NP), malate dehydrogenase (MD) and aspartate aminotransferase (AST).

### Random amplified polymorphic DNA (RAPD) analysis

Random DNA fragments can be amplified from cellular DNA using short primers; the resulting fragment patterns differ between species 21.

DNA was extracted from cells of the MCB and amplified by PCR using random primers. DNA from HEK 293 cells and from the RCB was used as positive controls; DNA from murine, ovine, monkey [foetal rhesus monkey kidney (FRhK-4)] and hamster (CHO) cells served as negative controls. PCR products were analysed by agarose gel electrophoresis and visualised under UV light.

### Safety characterisation of cell banks and production cells

In agreement with current guidelines, MCB, WCB and end of production cells (EPC) were analysed for identity by isoenzyme and RAPD analysis as described earlier. MCB, WCB and EPC were moreover tested for sterility, mycoplasma and adventitious agents. Furthermore, unprocessed bulk harvests (BH) were also included in the characterisation. This BH testing was extended compared with the BH release testing also described in the Results section.

A cell-based assay was used for mycoplasma testing of the MCB, the WCB, EPC and BH, as recommended by versions 5 and 6 of the European Pharmacopoeia (Ph.Eur.2.6.7), which were current at the time the assays were performed.

Viral testing included *in vitro* assays in Vero, MRC5 and HEK 293 cells incubated for 28 d and *in vivo* assays in adult and suckling mice and embryonated eggs. *In vitro* screens for bovine and porcine viruses were also performed. PCR was used to screen for human viruses, adeno-associated virus (AAV)-2 and bovine polyoma virus, and quantitative fluorescent product-enhanced reverse transcriptase (QF-PERT) for retroviruses. Screening for retroviral-like particles in cells and culture supernatant was carried out by transmission electron microscopy (TEM); the mouse minute virus (MMV) infectivity assay evaluated both the presence of MMV and the capability of the cells to propagate MMV.

All assays used for viral testing were conducted in agreement with current guidelines on viral safety evaluation 22, 24. All analyses were performed by an accredited good laboratory practice (GLP)-/good manufacturing practice (GMP)-compliant contract laboratory.

### Safety characterisation of media and equipment

A GMP-compliant serum-free FreeStyle™ 293 expression medium was used for the generation of the cell line. A proprietary low-protein medium free of human or animal additives is employed in the production process. All chemicals are compliant with the European Pharmacopoeia, and all equipment and all processes are GMP-compliant. Suppliers have to certify that no animal-derived material has been used in the production of any raw materials employed in the manufacturing process, including chromatography media, the affinity ligand and filters.

### In-process control

Manufacturing is performed in classified facilities under GMP. Cell culture harvest is tested for bioburden, mycoplasma and adventitious viruses; acceptance criteria for further processing have been specified. Purification equipment is cleaned between runs following documented procedures and controlled for potential contamination. The final drug substance and drug product are tested for endotoxin, bioburden and sterility; defined acceptance criteria have to be met for release. All tests are compliant with standard methodology according to the European and US Pharmacopoeia.

### Purification process

A multistep purification process for human-cl rhFVIII has been developed to optimise the level of purity and pathogen safety. Chromatography resins and filters used are Capto MMC™, SP Sepharose FF®, FVIIISelect™, Q Sepharose FF®, Superdex 200 pg™ (all from GE Healthcare Life Sciences, Uppsala, Sweden), Sartobind® Q (Sartorius Stedim Nordic A/S, Taastrup, Denmark) and Planova 20N™ (N.V. Asahi Kasei Bioprocess Europe S.A., Brussels, Belgium).

### Quantification of residual DNA

Residual host DNA is determined by the Threshold™ DNA assay kit (Molecular Devices Limited, Wokingham, UK) according to manufacturer's instructions. The method uses a DNA-binding protein, which immobilises single-stranded DNA on a membrane, and an enzyme-linked anti-DNA antibody for detection. According to the manufacturer, the sensitivity of this assay allows the detection of 2 pg DNA per sample 25.

### E1A assay

DNA was extracted using the QIAamp® viral RNA Mini Kit (QIAgen Nordic, Sollentuna, Sweden) that copurifies DNA and RNA. Purified water was spiked with 1 ng HEK 293 DNA to assess extraction efficiency. In addition, 1000 copies of positive control DNA were used to spike aliquots of each sample to assess inhibition. qPCR analysis of E1A was performed at a contract laboratory using primers and probes specific for the E1A region of adenovirus 5; all samples, except the sentinel and the blank water control, were analysed in triplicates. The assay was validated according to ICH Q2 26.

### Validation of virus clearance capacity

According to current European guidelines, at least two viral inactivation/removal steps with different modes of action should be assessed 27, and also US guidelines call for the assessment of more than one step 28. Solvent/detergent (S/D) treatment and Planova 20N™ nanofiltration were chosen and investigated using a validated down-scale version of the manufacturing process.

To analyse the virus inactivation capacity of S/D treatment, appropriate in-process material was mixed with tri-n-butylphosphate (TnBP) and Octoxynol 9 and subsequently spiked with pseudorabies virus (PRV). Test samples were taken after 1, 5, 10, 20 and 30 min; the virus inactivation process was immediately terminated by diluting samples with cell culture medium. For the analysis of the virus removal capacity of Planova 20N™ nanofiltration, appropriate in-process material was spiked with porcine parvovirus (PPV). Nanofiltration was performed, and the filter was washed with buffer. Test samples were taken before and after nanofiltration. All test samples were analysed by means of infectivity assays. Vero and PK13 cells were used as indicator cells for PRV and PPV, respectively.

## Results

### Development of production cells

HEK 293 cells were originally established by Frank Graham in the laboratory of Alexander van der Eb 29–30. These cells have been widely accepted and used in academic research for more than 30 years, with documented excellent growth, transfection efficiency and satisfactory recombinant production yields. Their use as a commercial expression platform was considered after Invitrogen adapted the cell line in 2003 to suspension growth and in serum-free medium (HEK 293 F cells) 18.

HEK 293 F cells were chosen as a host cell line because the parental HEK 293 cells had been used extensively at Octapharma before, and optimised methods for transfection, selection, clonal isolation and expansion had already been developed and established by Octapharma. The fact that these cells grow in the absence of serum enabled the establishment of the expression cell line in the absence of any material of animal origin. Cell culture parameters were optimised for industrial use, for example the splitting rhythm to ensure a long-term, stable culture over years; cryopreservation methods (as almost all known methods at the time required serum); the vector backbone, to ensure good recombinant productivity; and the cultivation process, to achieve maintained maximal protein yields. Moreover, a proprietary medium without animal-derived components was developed to ensure the stability of production quality and supply and to minimise pathogen infectivity risk.

**Figure 1:**
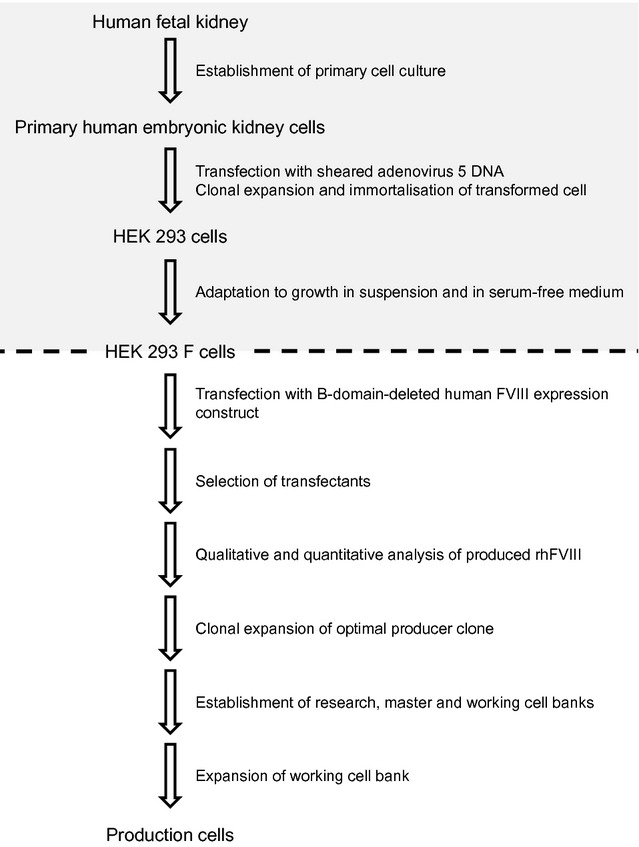
Development of the production cell line for human-cl rhFVIII. A culture of primary cells established from a kidney of a female human foetus was transfected with sheared adenovirus 5 DNA. A single transformed cell was isolated, expanded and immortalised, giving rise to the human embryonic kidney (HEK) 293 cell line. Adaptation to growth in suspension and in serum-free medium produced the HEK 293 F cell line. This cell line was stably transfected with an expression plasmid encoding a B-domain-deleted human FVIII protein. Transfectants were selected, expanded and optimised for maximal expression of the human FVIII protein. All steps below the dotted line were performed by Octapharma.

Stable transfection and selection, as described in the Materials and methods section, led to a clone that produced fully functional rhFVIII (Sandberg H, Kannicht C, Stenlund P, Dadaian M, Oswaldsson U, Cordula C, Walter O, manuscript submitted for publication). The clone was expanded to establish a cell bank system from which all production cells were generated (Fig. 1).

**Figure 2:**
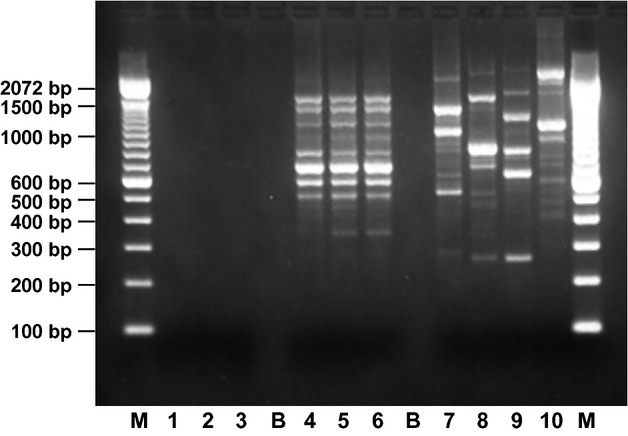
Results of RAPD analysis. RAPD banding patterns for MCB and controls. M, molecular mass standard (100-bp ladder); B, blank; 1, sentinel to assess airborne contamination; 2, water to assess reagent contamination; 3, extraction control; 4, human embryonic kidney (HEK) 293 DNA, 5, RCB DNA; 6, MCB DNA; 7, mouse DNA; 8, ovine DNA; 9, monkey DNA (FRhK-4); 10, hamster DNA (CHO). RAPD, random amplified polymorphic DNA; MCB, master cell bank; RCB, research cell bank; FRhK-4, foetal rhesus monkey kidney; CHO, Chinese hamster ovary.

The appropriateness of this cell line for rhFVIII production was fully investigated by analysing the viability of the MCB after storage under defined conditions and the genetic stability of the expression construct in cells at the limit of *in vitro* cell age, according to current regulatory requirements (data not shown) 23. For the MCB, human origin and identity and the absence of any non-human contamination were confirmed at the protein and DNA levels by isoenzyme analysis and RAPD, respectively, as recommended by current regulatory guidance 23. The isoenzyme migration pattern was consistent with that of a human control sample and with that of HEK 293 F cells from which the production cell line was derived (Table 1). RAPD analysis yielded almost identical fragment patterns to those obtained with HEK 293 DNA and DNA extracted from the MCB and RCB, whereas no similarity in banding patterns was observed with mouse, ovine, monkey or hamster DNA (Fig. 2).

**Table 1:** Results of the isoenzyme analysis

Enzyme	Expected migration (mm)	Actual migration assay control (mm)	Actual migration MCB (mm)^1^	Actual migration HEK 293 F (mm)^1^
NP	12.8	15.0	15.0	15.0
MD	8.3	10.0	10.0	10.0
AST	15.0	16.0	16.0	16.0

MCB, master cell bank; NP, nucleoside phosphorylase; MD, malate dehydrogenase; AST, aspartate aminotransferase.

1 The samples of the MCB and human embryonic kidney (HEK) 293 F cells were not run together in the same experiment; however, owing to the standardisation of the assay, the assay migration control showed identical values in both runs. Results for the MCB and HEK 293 F cells are therefore presented side by side.

### Safety characterisation of cell banks and production cells

Table 2 shows the results of all tests performed on the MCB, WCB, EPC and BH. Isoenzyme and RAPD analysis confirmed human origin at the protein and DNA levels for all cells tested. All samples were free of microbiological organisms (bacteria and fungi) and mycoplasma.

**Table 2:** Overview of tests performed and results obtained

	MCB	WCB	EPC	BH
Identity				
Isoenzyme analysis	HEK 293	–	–	–
RAPD	HEK 293	HEK 293	HEK 293	–
Microbiological tests				
Sterility	Complies	Complies	Complies	Complies
Mycoplasma	Negative	Negative	Negative	Negative
Viral tests				
*In vitro* assay for the detection of adventitious viruses (28 d, three detector cell lines: Vero, MRC5 and 293)	Negative	–	Negative	Negative
*In vivo* assay for the detection of adventitious viruses (adult and suckling mice, embryonated eggs)	Negative	–	Negative	Negative
QF-PERT	Negative	–	Negative	Negative
PCR screen for human viruses (HIV 1/2, HTLV 1/2, CMV, EBV, HHV 6/7/8, HBV, HCV, B19, HPV, HPyV)	Negative	–	–	–
PCR screen for AAV-2	Negative	Negative	Negative	Negative
TEM	No virus detected	–	No virus detected	No virus detected
MMV infectivity assay	Negative	–	–	–
*In vitro* bovine virus screen (BVDV, BAV, BRSV, BPV, REO3, BTV, RV)	Negative	–	–	–
PCR screen for bovine polyoma virus	Negative	–	–	–
*In vitro* porcine virus screen (PPV, PAV, TGE, HEV)	Negative	–	–	–

MCB, master cell bank; WCB, working cell bank; EPC, end of production cells; BH, bulk harvest; RAPD, random amplified polymorphic DNA; QF-PERT, quantitative fluorescent product-enhanced reverse transcriptase; PCR, polymerase chain reaction; HEK, human embryonic kidney; HIV, human immunodeficiency virus; HTLV, human T-cell lymphotropic virus; CMV, cytomegalovirus; EBV, Epstein–Barr virus, HHV, human herpes virus; HBV, hepatitis B virus; HCV, hepatitis C virus; B19, human parvovirus B19; HPV, human papilloma virus; HPyV, human polyoma viruses JC and BK; AAV, adeno-associated virus; TEM, transmission electron microscopy; MMV, mouse minute virus; BVDV, bovine viral diarrhoea virus; BAV, bovine adenovirus; BRSV, bovine respiratory syncytial virus; BPV, bovine parvovirus; REO, reovirus; BTV, bluetongue virus; RV, rabies virus; PPV, porcine parvovirus; PAV, porcine adenovirus; TGE, transmissible gastroenteritis virus; HEV, haemagglutinating encephalitis virus.

– indicates tests have not been performed.

Extensive tests for viruses demonstrated the absence of virus and virus- or retrovirus-like particles. The cell line was therefore considered suitable for the production of recombinant proteins and has been classified as case A according to ICH Q5A. This is the lowest risk classification; both BHK and CHO cells are classified as case B 24.

### Acceptance and release criteria

Acceptance criteria for adventitious agents in BH are listed in Table 3. Table 4 provides the release criteria for the drug substance and the final product. The BH has to be free of mycoplasma and adventitious viruses in order for the cell culture harvest to be acceptable. There are defined limits for the bioburden of the BH and the drug substance, and for the endotoxin content of the drug substance and final product. The final product must also be sterile.

**Table 3:** Acceptance criteria for adventitious agents in BH

Test	Method	Acceptance criteria
Bioburden	Ph.Eur., USP	<1 CFU/5 mL
Mycoplasma	Ph.Eur. 6.0, 2.6.7	Negative
*In vitro* adventitious virus	28 d, Vero, MRC5 and 293 cells	Negative

BH, bulk harvest; Ph.Eur., European Pharmacopoeia; USP, United States Pharmacopeia; CFU, colony-forming unit.

**Table 4:** Microbial release criteria for drug substance and final product

	Test	Method	Release criteria
Drug substance	Endotoxin	LAL test, Ph. Eur, USP	<3 EU/100 IU VIII:C
	Bioburden	Membrane on agar method	≤10 CFU/mL
Drug product	Endotoxin	Ph. Eur, USP	<3 EU/100 IU VIII:C
	Sterility	Ph. Eur, USP	Approved

LAL, *Limulus* amoebocyte lysate; Ph.Eur., European Pharmacopoeia; USP, United States Pharmacopeia; EU, endotoxin units; IU, international unit; CFU, colony-forming unit.

### Purification process

**Figure 3:**
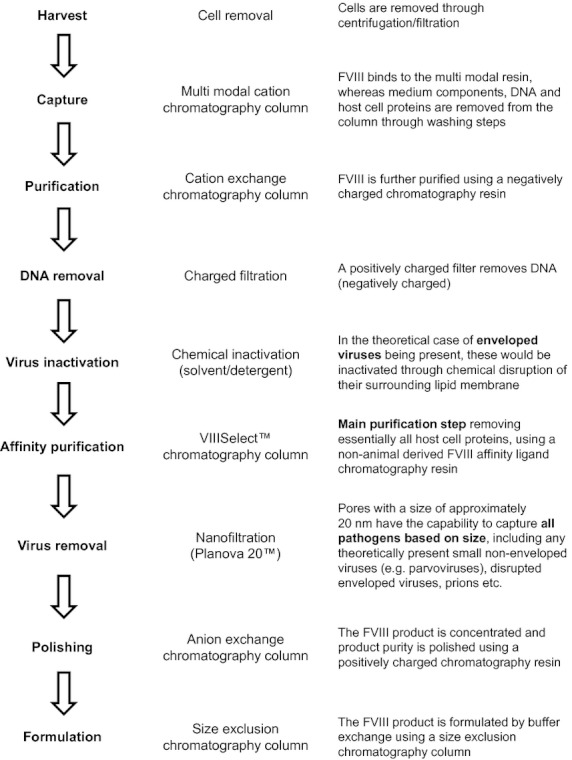
Purification of human-cl rhFVIII. Detailed description of the function of the purification steps used in the manufacturing of human-cl rhFVIII; brand names of chromatography media and filters are provided where appropriate.

The purification process in the production of human-cl rhFVIII is illustrated in Fig. 3. A total of one centrifugation, two filtration and five chromatography steps are employed to remove host cells, medium components, chemicals used during purification, DNA and proteins. The key purification step uses a non-animal-derived affinity ligand that is produced in yeast cells and is specific for FVIII. Two dedicated virus clearance steps, S/D treatment and nanofiltration through a 20 nm pore size filter, are included to ensure inactivation/removal of potentially present enveloped and non-enveloped viruses.

### Quantification of residual DNA

The kit used for the quantification of residual DNA ensures the detection of all single-stranded DNA molecules ≥100 base pairs (bp), that is, of fragments well below the minimum size of a functional gene, which, based on current evidence, is estimated to be ∼200 bp 31. Residual DNA has been shown to be below 10 pg/1000 IU in the final product (Sandberg H, Kannicht C, Stenlund P, Dadaian M, Oswaldsson U, Cordula C, Walter O, and Kannicht C, Ramström M, Kohla G, Tiemeyer M, Casademunt E, Walter O, Sandberg H, manuscripts submitted for publication).

Eluates from the gel filtration and the DNA removal steps were analysed for the presence of adenovirus 5 E1A DNA by qPCR. Spiking assays demonstrated the absence of inhibitory substances interfering with PCR amplification from all samples. No amplification was detected in any of the replicates for either sample, indicating that, within the limits of the sensitivity of the assay, the eluates were negative for the presence of E1A sequences (Table 5).

**Table 5:** Removal of E1A DNA by the purification process

Sample	*n*	Mean C_*T*_ value	Mean copies/reaction
Sentinel control	2	40.0	0
Blank water control	2	40.0	0
Extraction control	3	40.0	0
Spike recovery control (purified water + 1 ng HEK 293 DNA)	3	31.02	1443
HEK 293 DNA (1 ng)	3	29.89	3023
**DNA removal eluate**^1^	3	40.0	0
**Gel filtration eluate**^2^	3	40.0	0
**DNA removal eluate** + 1000 copies^1^	3	31.59	1012
**Gel filtration eluate** + 1000 copies^2^	3	31.64	966

Samples marked in bold text are in-process samples taken after the DNA removal step^1^ and the gel filtration step^2^, respectively (see Fig. 3 for the order of steps in the purification process). HEK, human embryonic kidney.

### Virus inactivation and removal

The model viruses initially chosen to assess S/D treatment and nanofiltration were PRV and PPV, respectively. PRV is generally considered a model for the hepatitis B virus 32; PPV is accepted as a model for human parvovirus B19 33. Both these viruses are considered appropriate model viruses in current regulatory guidance 28, 34.

**Figure 4:**
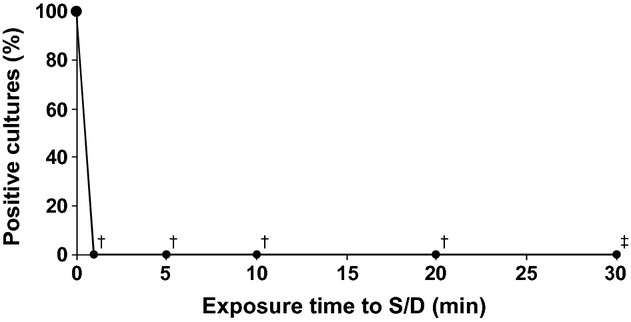
Kinetics of PRV inactivation by S/D treatment. In-process material was mixed with TnBP and Octoxynol 9 and subsequently spiked with PRV. Samples were taken at the indicated time points and analysed by infectivity assays in Vero cells (*n* = 2). ^†^Below detection limit 1; ^‡^Below detection limit 2. PRV, pseudorabies virus; S/D, solvent/detergent; TnBP, tri-n-butylphosphate.

**Figure 5:**
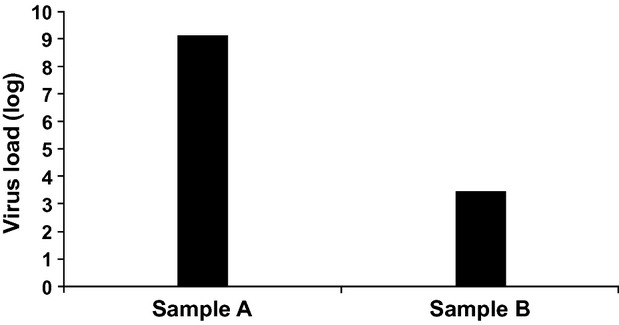
PPV removal by Planova 20N™ nanofiltration. PPV-spiked in-process material was subjected to Planova 20N™ nanofiltration, and the filter was washed with buffer. Samples (sample A: prior to nanofiltration; sample B: after nanofiltration and postwash) were analysed by infectivity assays in PK13 cells (*n* = 2). PPV, porcine parvovirus.

The kinetics of virus inactivation by S/D treatment are shown in Fig. 4, and virus removal by the nanofiltration step is illustrated in Fig. 5. After S/D treatment, no viruses were detectable at any time points tested, despite an improved 15-fold higher detection limit in the sample taken after 30 min. The virus reduction factor after 30 min was ≥6.29 ± 0.24 log and ≥6.29 ± 0.28 log [mean ± 95% confidence interval (CI)] in two independent experiments.

Excellent virus removal was achieved with Planova 20N™ nanofiltration, resulting in virus reduction factors of 5.45 ± 0.34 log and 5.69 ± 0.32 log (mean ± 95% CI) in two independent experiments.

## Discussion

This article summarises the manufacturing process and safety profile of the first rhFVIII produced in a human cell line. The production cell line was established based on a current cell line widely used for decades, which was then transfected with an expression plasmid for rhFVIII production. The human identity of these cells was confirmed by isoenzyme analysis and RAPD; extensive analyses showed no evidence of the presence of any adventitious agents. Moreover, no animal- or human-derived materials were used in the manufacturing process, which minimised the immunogenic potential and the risk of introduction of adventitious agents. The purification process included two dedicated virus inactivation/removal steps, S/D treatment and nanofiltration. Multiple stringent purification steps efficiently eliminated DNA and protein derived from the host cells. In accordance with GMP, the process and all equipment were closely monitored to ensure product quality.

From a pathogenic point of view, rhFVIII products are extremely safe; no cases of viral transmission with these products have been reported in over 20 years of commercial use. Extensive analyses according to current regulations have not been able to detect any virus or virus-like particles in the MCB, WCB or EPCs used for the production of human-cl rhFVIII. This is the first rhFVIII product manufactured in a human cell line without the use of animal- or human-derived materials. Therefore, the risk of viral contamination of the final product can be considered the lowest in the class and should be negligible. In the theoretical case of viral contamination, two dedicated state-of-the-art virus clearance methods integrated in the manufacturing process reliably destroy or remove any potential viruses, both enveloped and non-enveloped.

S/D treatment is accepted worldwide as an effective, robust and simple method that efficiently inactivates all enveloped viruses 35 and is considered the gold standard in this respect. Of note, no proven transmission of an enveloped virus has been reported because S/D treatment was introduced into the manufacturing process for plasma-derived medicinal products 35. An effective method for the removal even of small, non-enveloped viruses is nanofiltration: complete elimination by size exclusion of a number of viruses has been demonstrated for Planova 20N™ 36, the 20 nm pore size filter used for the nanofiltration step in the manufacturing of human-cl FVIII.

As stipulated by regulatory authorities 22, 24, the virus clearance capacities of two orthogonal process steps have been determined using PRV (a robust model for enveloped viruses) to assess the S/D treatment and PPV [a model for the smallest and most robust non-enveloped viruses 37] to validate the Planova 20N™ nanofilter. Results confirmed that both process steps were capable of efficiently clearing even high virus loads. The fact that even PPV, a particularly challenging virus because of its small size and highly robust nature, can be reliably removed indicates that the manufacture of human-cl rhFVIII is a powerful process to inactivate/remove any potential virus contaminations, allowing the final product to set new standards in pathogen safety. In comparison, the purification processes for other approved rhFVIII products either do not include nanofiltration as a virus removal step, or use nanofilters with bigger pore sizes (Table 6).

**Table 6:** Comparison of different safety-related topics for rhFVIII products

Topic	Advate	Kogenate	Xyntha	Refacto	Human-cl rhFVIII
Cell line	Rodent (CHO) 4	Rodent (BHK) 51	Rodent (CHO) 43	Rodent (CHO) 52	Human (HEK 293 F)
Immunogenic potential (glycosylation)^1^	+	+	+	+	–
DNA, pg/1000 IU FVIII	<10 51	<10 51	<10 43	<10 51	<10
Affinity chromatography rodent antibodies	Yes 4	Yes 51	No^2^ ^43^	Yes 52	No^3^
Immunogenic potential (rodent host cell proteins^4^)	Yes 4	Yes 5	Yes 6	Yes 6	No
Virus/prion^5^ removal by nanofiltration	No 4	No 51	Yes 43; 35 nm pores	No 52	Yes; 20 nm pores
Virus inactivation by S/D treatment	Yes 4	Yes 51	Yes 43	Yes 52	Yes
Expected prion reduction in purification process	No data	No data	>9 log 43	>9 log 43	>>9 log^5^

rhFVIII, recombinant human clotting factor VIII; BHK, baby hamster kidney cells; CHO, Chinese hamster ovary; IU, international units; S/D, solvent/detergent.

1 rhFVIII derived from human human embryonic kidney (HEK) 293 F cells are devoid of antigenic sialic acids of *N*-glycolylneuraminic acid type and antigenic Galα1,3Gal carbohydrate, which can be found in rhFVIII produced in hamster cell lines (Kannicht C, Ramström M, Kohla G, Tiemeyer M, Casademunt E, Walter O, Sandberg H, manuscript submitted for publication).

2 Peptide affinity ligand (43).

3 13 kD antibody fragment affinity ligand produced in *Saccharomyces cerevisiae* (53).

4 Trace amounts of rodent host cell proteins and rodent monoclonal antibodies (from affinity purification) in final product.

5 Prion removal potential for nanofilters with small pores (i.e. 20 nm) and three chromatography steps (Q Sepharose FF®, FVIIISelect™ and Capto MMC™.

Today, it is generally recognised that theoretical concerns regarding residual cellular DNA can be minimised by reducing the size and amount of DNA fragments in the final product 38–39. Regarding vaccines, the US Office of Vaccines Research and Review (OVRR)/Center for Biologics Evaluation and Research (CBER) has stated that ‘there is an extremely low probability that residual DNA from the adenovirus 5-transformed human cells could transfer traits that could induce neoplastic development’ in people receiving these vaccines 40. In line with these views, the Vaccine Cell Substrate conference in 2004 concluded that the evidence available at that time did not support the oncogenicity of residual host DNA 39. Human-cl rhFVIII is not a vaccine, but a highly purified product; however, also for the production of biologics such as human-cl rhFVIII, it is recommended that the level of residual host DNA in the final product should be as low as possible 41. The production process for human-cl rhFVIII has been shown to efficiently remove DNA from the product. Moreover, residual DNA content in the final product is routinely monitored using the Threshold (Molecular Devices Limited) method and has been found to be below 10 pg/1000 IU for the final product of human-cl rhFVIII (Sandberg H, Kannicht C, Stenlund P, Dadaian M, Oswaldsson U, Cordula C, Walter O, and Kannicht C, Ramström M, Kohla G, Tiemeyer M, Casademunt E, Walter O, Sandberg H, manuscripts submitted for publication). This is well below the 10 ng/dose considered acceptable by the WHO 42 and corresponds to the level of purity achieved with other recombinant products 43.

Prion transmission via biologics produced in animal or human cell lines has never been reported so far; and even though some patients received plasma-derived blood products from donors who developed variant Creutzfeldt–Jakob disease after donation, there have been no proven transmissions to date 44. HEK 293 F cells express the non-pathogenic normal PrP protein, as do essentially all human and animal cell lines, including CHO and BHK cells used to produce rhFVIII (Octapharma, data on file). The level of expressed PrP protein in HEK 293 F cells is within a comparable range to those observed in the two hamster cell lines (data not shown).

PER.C6 cells have been approved by the FDA for the production of vaccines and therapeutic biologics. Of note, these cells were established by adenoviral transformation of human embryonic retina 45 and can be considered to be of neuronal origin. This highlights the minimal prion risk even when using cells derived from tissues with high levels of normal non-pathogenic PrP expression 46.

The purification process of human-cl rhFVIII has been designed to address the theoretical safety concern of prion contamination. The level of residual host cell protein is routinely checked in the final product, and release criteria have been defined to ensure a very low host cell protein level in all batches for clinical use (Sandberg H, Kannicht C, Stenlund P, Dadaian M, Oswaldsson U, Cordula C, Walter O, manuscript submitted for publication). The affinity step, VIIISelect™, is highly specific for FVIII and has been shown to reliably remove essentially all host cell proteins. The Capto MMC™ resin used for the mixed mode capture chromatography step also binds FVIII through multiple interactions, providing an additional tool for the removal of impurities. Preliminary data indicate that potentially present PrP is not able to bind to this chromatography resin and is therefore completely removed from the column prior to product elution (data not shown). Moreover, human-cl rhFVIII is the only rhFVIII to date that is purified using a 20 nm pore size filter (Planova 20N™) for nanofiltration, which has been demonstrated to remove prions from biological solutions 47–48. Infectivity and conversional activity have been reported to be highest in non-fibrillar prion particles of 17-27 nm diameter 49. This size corresponds well to that of PPV, which has been shown to be efficiently removed by the Planova 20™ filter 36.

In the purification process of a fourth-generation rhFVIII product that is already on the market, prion removal capacities of 3.8 logs and >5.2 logs have been demonstrated for the immunoaffinity step and the Q Sepharose FF® step, respectively, resulting in a total clearance of >9 log 50. Similar steps (affinity purification and Q Sepharose chromatography) are also present in the purification process for human-cl rhFVIII, and in addition, the nanofiltration and the Capto MMC™ step can be expected to contribute to prion reduction as well. Therefore, from a theoretical perspective, it is safe to assume that the total clearance will be superior to that reported for the other rhFVIII product mentioned earlier.

## Conclusions

The rationale behind a human host system, free from added animal- or human-derived materials, for producing rhFVIII is ultimately the reduction of immunogenic challenge to patients. A manufacturing process using a well-known cell line suitable for rhFVIII production was designed and developed for commercial use. Three complementary approaches were used to minimise the risk of potential contamination: thorough testing of cell lines and close control of raw materials, the capacity of the production process to eliminate potential contaminations and systematic analysis of the final product. The safety data obtained to date demonstrate that human-cl rhFVIII can be safely used in the clinic and that the manufacturing process sets new standards in terms of purity and pathogen safety.
